# Inhibition of antioxidant enzyme activities enhances carotenogenesis in microalga *Dactylococcus dissociatus* MT1

**DOI:** 10.3389/fbioe.2022.1014604

**Published:** 2022-09-23

**Authors:** Nour Elaimane Bouzidi, Samir Borhane Grama, Aboubakeur Essedik Khelef, Duanpeng Yang, Jian Li

**Affiliations:** ^1^ Laboratory of Natural Substances, Biomolecules and Biotechnological Applications, University of Oum El Bouaghi, Oum El Bouaghi, Algeria; ^2^ College of Chemical and Biological Engineering, Panzhihua University, Panzhihua, China

**Keywords:** microalgae, carotenoids, antioxidant enzymes, reactive oxygen species, enzyme inhibitors

## Abstract

Microalgal biotechnology has become a promising field of research for the production of valuable, sustainable and environmentally friendly byproducts, especially for carotenoids. Bulk accumulation of secondary carotenoids in microalgae are mostly induced by oxidative stress of cells. In this research, we investigated the effects of antioxidant enzyme activity inhibition on carotenogenesis in a microalga *Dactylococcus dissociatus* MT1. The activities of four major antioxidant enzyme families, namely superoxide dismutase (SOD), catalases (CAT), glutathione peroxydases (GPX) and ascorbate perxodases (APX), were inhibited by relevant inhibitors during the stressed cultivation of *D. dissociatus* to observe the effects on carotenogensis. A 91% decrease in activity was observed for CAT, comparing with controls without any inhibitors added, followed by 65%, 61%, and 47% for the enzymes SOD, APX, and GPX, respectively. Concomitantly, it was found that this partial inhibition had substantial influences on the accumulation of carotenoids, with the highest production levels obtained in CAT inhibition conditions and an increase of 2.6 times of carotenoid concentration observed, comparing with control cultivation conditions. We conclude that the modulation of antioxidant enzyme activities could lead to the overproduction of carotenoids in this microalgal cell culture, and we expect that this novel approach of optimizing carotenogenesis processes for *D. dissociatus* cell cultures could be transferrable to other cell culture systems and might have an important impact on the carotenoid production industry.

## 1 Introduction

Carotenoids are pigmented terpenoids, which can be synthesized by microalgae or plants as accessory pigments for photosynthetic apparatus or as secondary metabolites in response to intracellular oxidative stress. Carotenoids are strong antioxidants with application in the food, cosmetic, aquaculture and pharmaceutical industries, and two of carotenoids (astaxanthin and adonixanthin) accumulated in the strain of *Dactylococcus dissociatus* MT1 isolated from the Sahara Desert of Algeria have been shown to have anti-tumor and anti-carcinogenic properties Grama et al. (2014a); Maoka et al. (2013). As a result, it has recently been suggested that certain carotenoids could be used as chemotherapeutic or anti-angiogenic agents Ganesan et al. (2013).

Although currently the majority of carotenoids are produced by chemical synthesis, carotenoid production from microalgae has been of increasing interests recently. Firstly, chemical carotenoids are synthesized from petrochemical compounds, which is not sustainable. Secondly due to health and environmental concerns, chemical carotenoids are restricted from human and some animal consumption, contrary to the natural carotenoids which has already been commercialized for food supplement market (ex. BioAstinm, Nutrex-Hawaii) Li et al. (2011). Utilization of synthetic antioxidants has declined because of their supposed carcinogenic activity as well as the widespread rejection of synthetic additives in the diet by consumers?. Alternatively, natural carotenoids can be obtained from microalgal cell cultures, which is particularly promising because of their environment-friendly properties and carbon-negative nature.

The bulk accumulation of secondary carotenoids is a physiological response to counter the deleterious effects of intracellular oxidative stress caused by reactive oxygen species (ROS), such as the superoxide anion or H_2_O_2_. ROS can be produced when linear electron flux (LEF) in the photosynthetic electron transport chain (ETS) is perturbed at the level of the cytochromes or ferredoxin, or if the LEF is blocked by an excess of terminal reducing equivalent (Solovchenko, 2013). ROS as the by-products of oxidative metabolism are produced in aerobic organisms, which is an ubiquitous phenomena and can be mitigated by antioxidant systems of cells. However, at high production rates, these species may be very deleterious to living cells and organisms. When the levels of these ROS surpass the antioxidant system’s ability to process them, the damage occurs Pinto et al. (2003). Likewise, with increasing intracellular oxygen concentration, the O_2_ begins to outcompete CO_2_ at the active site of the enzyme RuBisCo, which induces photorespiration and ultimately results in the production of H_2_O_2_, a powerful oxidant that is toxic to the cells. The decomposition of H_2_O_2_ is catalysed by the activity of ascorbate peroxidase (APX) or by catalase (CAT), both of which are hemoproteins. In addition to the decomposition of ROS by enzymatic catalysis, the carotenoids also possess high antioxidant activity and are efficient scavengers of ROS Edge et al. (1997). Not surprisingly, the level of intracellular ROS can be related to both the activity of the antioxidant enzymes and the production of the carotenoids Michelet et al. (2013). It should be noted that oxidative damage may result from either the rise of reactive species (ROS) in the cell or the decrease in cell efficiency to eliminate them Arunakumara and Zhang (2008).

In the plant system, including microalgae, reactive oxygen species (ROS) are consistently generated by the consequent leakage of electrons to molecular oxygen through the electron transport activities of various organelles including chloroplasts, mitochondria, and the plasma membrane Foyer et al. (1997); Mallick and Mohn (2000). In addition, both biotic and abiotic stresses may lead to additional enhancement of ROS levels Rabbani et al. (1998). ROS are highly toxic and result from the partial reduction of harmless molecular oxygen. Reactive forms of oxygen include the superoxide radicals ( $O_{2}^{-}$ ), the hydroxyl radical (OH^−^) and hydrogen peroxide (H_2_O_2_). All these components may interact with particular biomolecules, affecting or inhibiting the biochemical functions as well as leading to oxidative degradation of cellular elements including DNA, proteins, lipids and pigments. ROS are also involved in the induction of stress tolerance and act as cytotoxic compounds Mallick and Mohn (2000); Park et al. (2008). As an example, the chloroplasts of algae are comprised of a complex system of membranes with high levels of polyunsaturated fatty acids, all of which could potentially be affected by peroxidation Halliwell and Gutteridge (2015). These hazardous compounds are generated at three principal locations throughout the photosynthetic process: the PSII LHC, the PSII reaction center and the PSI acceptor side Hajiboland (2014).

The current state of the art for the induction of these stresses for the purpose of secondary carotenogenesis is to combine nutritional stress with oversaturating light intensity Solovchenko (2013). However, it is difficult and often impossible to impose oversaturating light intensities on dense cell cultures of phototrophs, which are necessary for an economically viable process. Indeed, the bottleneck of phototrophic bioprocesses is the penetration of photons into the cell culture. Thus, the manipulation of the intracellular composition of ROS by affecting the intracellular redox state, either nutritionally or by targeting specific cell metabolisms are practical approaches to increase the production of secondary carotenoids while avoiding the bottleneck of photon penetration into the cell culture. Additionally, since secondary carotenoids are photosensitive, the high light intensities used to induce their production can also cause their degradation. Thus, the induction of secondary carotenogenesis through methods which reduce the dependence on saturating photon inputs could offer the benefit of increasing, rather than degrading, the target biomolecules.

The objective of this work was to study the enhancement of the biosynthesis of carotenoids in microalgae by manipulating the cellular ROS metabolism of the algal cells. Specifically, the induction of carotenogenesis were be realized firstly by conventional stressors (e.g., nitrogen deprivation, osmotic stress, high light intensity, etc.,) then these conventional methods will be combined with novel state-of-the-art bioprocess techniques which will increase the production of ROS by influencing parameters that have not been investigated, such as the inhibition of antioxidant enzyme activity. This novel and promising approach will develop microalgal bioprocess that avoids the necessity to deliver high light intensities into dense cell cultures and will therefore be more scalable and efficient than the conventional bioprocesses.

## 2 Materials and methods

### 2.1 Strain and media

*D. dissociatus* MT1 was field collected by the authors and identified as a strain of *Dactylococcus* by a phylogenetic analysis using the internal transcribed spacer (ITS-1 and ITS-2) sequences of the nuclear ribosomal RNA operon Grama et al. (2014a). The strain was kept in solid media, consisting of a medium 3N-BBM plus 7.5 g L^−1^ agar. The formula of the modified 3N-BBM was 8.8 mM NaNO_3_, 1.3 mM KH_2_PO_4_, 330 *μ*M K_2_HPO_4_, 430 *μ*M NaCl, 300 *μ*M MgSO_4_, 170 *μ*M CaCl_2_, 12 *μ*M EDTA, 2.2 *μ*M FeCl_3_, 2.0 *μ*M MnCl_2_, 220 nM ZnCl_2_, 99 nM Na_2_MoO_4_, and 51 nM CoCl_2_. Sterilization of the culture medium was performed by autoclaving for 20 min at 121°C and 1.6 bars.

### 2.2 Cell cultivation conditions

Liquid cultures of the algal suspension have been performed in 500 ml Duran glass bottles with a culture volume of 350 ml. The cultures were aerated by bubbling filtered air at room temperature and under continuous irradiation of 60 *μ*E m^−2^ s^−1^ supplied by cool white lamps. Exponential phase precultures were used for inoculation of the growth phase cultures.

The same procedures, growth and cultivation conditions for the precultures cultivations were also used for growth phase cultures, combining 50 ml of precultures with 350 ml of 3N-BBM. The cultures were grown in triplicate with an initial algal density in each flask of 0.21 ± 0.09 g L^−1^. The green phase cultivation was conducted over a period of 6 days.

After the green phase, the cells were separated from the culture medium and resuspended to the stress medium, which was identical to 3N-BBM except not containing any nitrate. In a previous study, the monod half-saturation constant Ek (*μ*E m^−2^ s^−1^) for the strain *D. dissociatus* MT1 was determined to be 110 *μ*E m^−2^ s^−1^. For this reason, the illumination was doubled to reach an intensity of 220 *μ*E m^−2^ s^−1^ for stressed cultivation. In this condition, an additional light-induced stress arose as a result of oversaturation of the photosystem at light intensities above the Ek Grama et al. (2014a).

### 2.3 Enzyme inhibitor addition

To differentiate the inhibition effects of antioxidant enzymes on the production of carotenoids, various inhibitors were added aseptically to the culture in the day 1 of stress phase cultivation, depending on the targeted enzymes. Addition of 2.5 mM H_2_O_2_ for the inhibition of SOD, addition of 68.4 mM NaCl for the inhibition of CAT, addition of 54 *μ*M zinc for the inhibition of GPX and addition of 10 *μ*M copper for the inhibition of APX were applied. A cell culture without any enzyme inhibitor was used as the control. The experiment runs were labelled as I-SOD, I-CAT, I-GPX and I-APX culture, for the culture with inhibitors of superoxide dismutase, catalase, glutathione peroxydase and ascorbate peroxidase respectively. The cultures were grown in triplicate with an initial algal density in each flask of at 0.34 ± 0.09 g L^−1^, and the stressed cultivation experiments were conducted over a period of 9 days.

### 2.4 Analytical techniques

#### 2.4.1 Cell culture density

In order to measure the density of dried cell mass in culture cells, 10 ml of the cell suspension were initially centrifuged in glass centrifuge tubes at 2000 g for 10 min to harvest the cell mass. The supernatant was decanted, and the cells were resuspended in 30 ml of distilled water and re-centrifuged for 10 min at 2000 g. The supernatant was then decanted, and the cell pellets were then transferred into pre-dried and pre-weighed aluminium dishes and dried for 24 h at 80°C before being reweighed. All measurements were conducted in triplicate Grama et al. (2014a).

#### 2.4.2 Nitrate analysis

Cells were removed from the supernatant (1.0 ml of culture) using centrifugation in 2.0 ml Eppendorf tubes at 5,000 g for 5 min. The supernatant was used for nitrate analysis, and the N $O_{3}^{-}$ content was subsequently determined at the beginning and end of the growth phase for each culture using a method based on UV absorption Rice et al. (2012). The measurements were conducted in triplicate.

#### 2.4.3 Pigment determination

Pigments were extracted by methanol. For 1.0 ml microalgal cell culture, 5 ml of methanol was added, mixed at room temperature for 5 min and then centrifuged at 2000 g for 1 min. The supernatant was kept for OD measurements, and the pellet was undergone further extraction by adding 5 ml of methanol until pellet turning white. The OD was measured with a UV-Visible spectrophotometer. The amounts of chlorophylls and total carotenoids were estimated using the equations below Lichtenthaler (1987):

[Cha] = 16.72 A_665.2_–9.16 A_652.4_

[Chb] = 34.09 A_652.4_–15.28 A_665.2_

[T—Car] = (1000 A_470_–1.63 [Cha]—104.96 [Chb])/221.

Where [Cha] [Chb] are the concentrations of chlorophylls a and b; [T—Car] is the concentration of total carotenoids. The final concentration of values pigments were normalizded to 1 L of cell culture and expressed in mg L^−1^. The measurements were conducted in triplicate.

#### 2.4.4 Lipid analysis

Lipids were extracted with modified Folch extraction procedure Folch et al. (1957) and the sulfo-phospho-vanillin (SPV) quantitation method Byreddy et al. (2016). The lipid content was determined by comparison to a calibration curve prepared from the standard addition of aliquots of 5, 10, 20, and 50 mg of high-purity soybean oil from a 100 mg ml^−1^ soy oil solution. All measurements were done in triplicate.

#### 2.4.5 Enzymatic activity measurement

50 ml of cell cultures were centrifuged, and the cells were re-suspended in sodium phosphate buffer (pH 7.0) as samples for analysis. Samples of cells were homogenized by an ultrasonic cell pulverizer at 220 W for a total duration of 5 min (ultrasonic time: 5 s; rest time: 10 s). The homogenate was centrifuged at 12,000 g for 15 min at 4°C. The supernatant was stored as an aliquot for enzyme estimation. Catalase (CAT) activity was determined spectrophotometrically, and the decomposition of H_2_O_2_ was monitored by the decline in absorbance at 240 nm Rao et al. (1996). Superoxide dismutase activity (SOD) was determined by the method of Beauchamp and Fridovich Beauchamp and Fridovich (1971) through measuring its ability to inhibit the photochemical reduction of nitrobluetetrazolium (NBT). Ascorbate peroxidase (APX) activity was calculated by the method of Nakano and Asada Nakano and Asada (1981), using ascorbate as a substrate, and the decrease in absorbance at 290 nm was measured. Glutathione peroxidase (GPX) activity was tested using added glutathione. The concentration of reduced glutathione (GSH) was determined spectrophotometrically with dithiontrobenzoic acid (DTNB) at 412 nm Rijstenbil et al. (1994). Protein concentration was determined using the standard Bradford colorimetric assay, with bovine serum albumin as the standards Bradford (1976). Taken together, the enzyme activities were normalized to the total protein and biomass concentrations. All measurements were performed in triplicate.

## 3 Results and discussion

### 3.1 Cell growth and carotenogenesis

**FIGURE 1:**
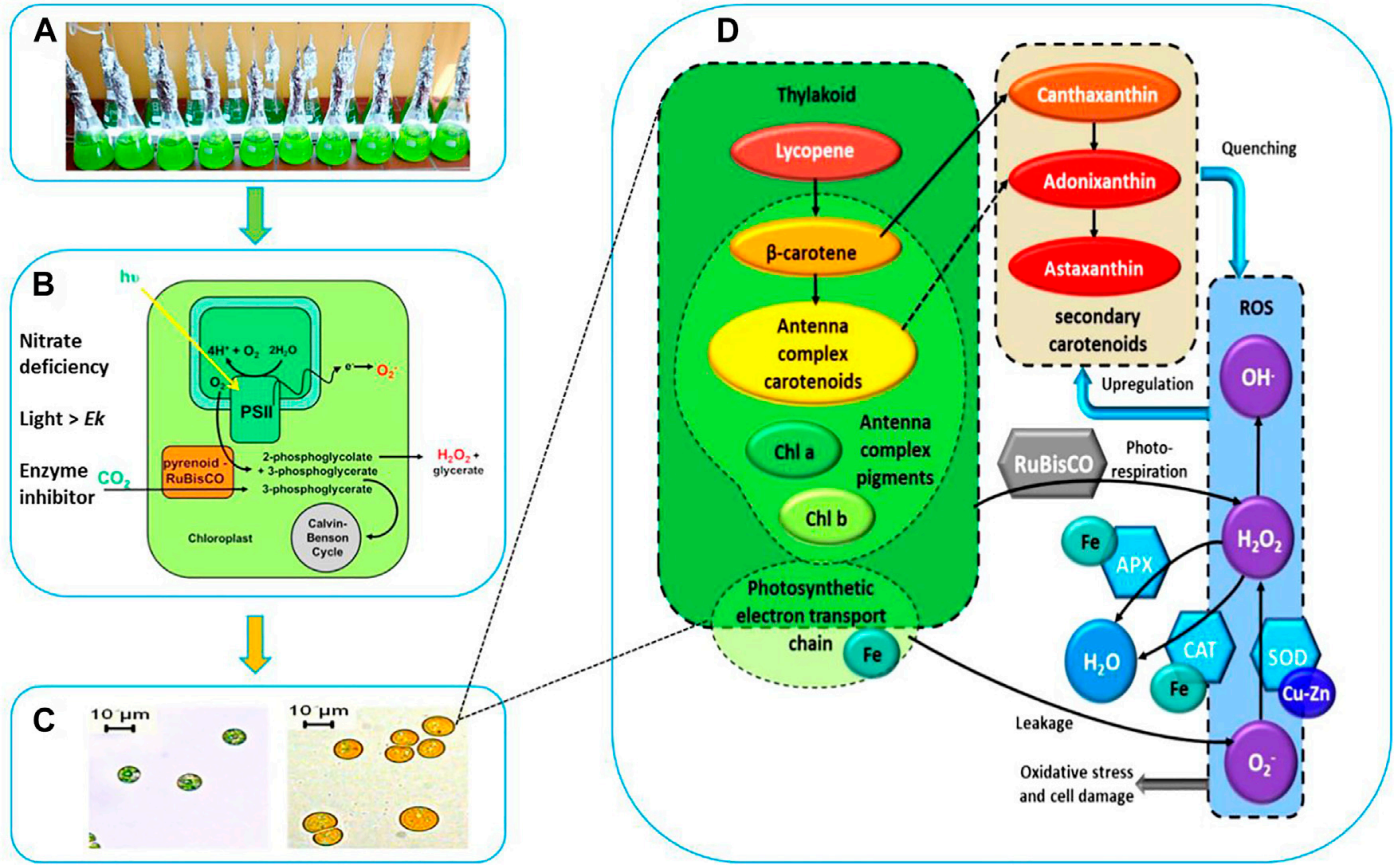
Schematic Representation of Stressed Cultivation with Enzyme Inhibitor Addition. **(A)** Stressed cell cultivation at T = 0 (inoculation). **(B)** Stressed conditions to induce carotenogenesis. **(C)** Microscopic observation of cell morphology and cellular pigments evolution. **(D)** Proposed interaction between the cofactors (Fe, Cu), antioxidant enzymes catalase (CAT), superoxide dismutase (SOD), ascorbate peroxidase (APX), the reactive oxygen species (ROS), the photosystem and the high-valued secondary carotenoids (canthaxanthin, astaxanthin, and adonixanthin).

Green growth phase cell culture of *D. dissociatus* MT1 were prepared for the carotenogenesis experimental runs. Under our experimental conditions, the cultivation of *D. dissociatus* MT1 was firstly cultivated for 6 days under nutrient replete conditions to accumulate green biomass and further cultivated under nitrogen deplete conditions to induce carotenogenesis (Figures 1A,B). At the end of the green phase cultivation, the concentration of N $O_{3}^{-}$ was decreased to 373.41 ± 20.74 mg L^−1^, which represented a 40% decrease of the initial concentration. Nitrogen is one of the critical nutrients in the microalgal metabolic cycles. This first step cultivation was carried out in order to obtain algal biomass that would be used for the second phase cultivation, namely the stress phase cultivation. The dry cell weight density at the start of the cultivation was 0.21 ± 0.09 g L^−1^ and increased up to 0.95 ± 0.12, which was a result of cell multiplication and individual growth. This increase reflected a cell growth characteristic of this stage. This growth of cell cultures was also accompanied by an increase in pH. After the first day of cultivation, the pH in the cell cultures increased from pH = 7.2 to roughly pH = 8.8. During the growth of photosynthetic microorganisms, the evolution of pH values cultivation may be related mostly to the relative rates of CO_2_ uptake by photosynthesis and supply by air bubbling. Photosynthesis consumes CO_2_ and subsequently shifts the equilibrium toward a higher pH. The pH increases of cell cultures indicated active phototrophic growth and insufficient CO_2_ supply to the cell culture.

At the end of the green growth phase, the residue nitrogen was removed from the cell culture by centrifugation, and the biomass was used to inoculate stress phase cultivation. The stress culture media without nitrogen were supplemented with a variety of antioxidant enzyme inhibitors individually for targeted enzymes. A control culture without inhibitor was performed to compare the effects of different inhibitors on the algal culture during the carotenogenesis process. It is widely reported that the accumulation and the production of secondary carotenoids can occur in microalgae through a variety of combinations of stresses. The developing trends for major components and parameters characterizing this carotenogenesis phase cultivation are reported in Table 1.

**TABLE 1:** Dry weight, cha, chb, T-car and lipid concentrations between inoculation (T = 0) and Termination (day 9) during the carotenogenesis process for all cultures.

	Dry weight	Cha	Chb	T—Car	Lipids
	(g L^−1^)	(mg L^−1^)	(mg L^−1^)	(mg L^−1^)	(mg L^−1^)
T = 0	0.34 ± 0.09	6.61 ± 0.11	3.91 ± 0.07	1.35 ± 0.13	70.35 ± 13
Control	0.41 ± 0.12	2.71 ± 0.18	0.16 ± 0.07	2.08 ± 0.26	170.32 ± 25
I-SOD	0.72 ± 0.15	0.66 ± 0.11	0.14 ± 0.05	3.94 ± 0.32	308.64 ± 26
I-CAT	0.88 ± 0.12	0.76 ± 0.23	0.11 ± 0.04	5.46 ± 0.45	431.58 ± 66
I-GPX	0.57 ± 0.07	0.77 ± 0.08	0.11 ± 0.02	3.29 ± 0.21	240.69 ± 39
I-APX	0.55 ± 0.08	0.74 ± 0.21	0.13 ± 0.03	3.22 ± 0.51	234.81 ± 21

In our work we hypothesized that the inhibition of antioxidant enzymes, which constituted the first line of defense against ROS, would lead to higher levels of these radicals in algal cells, which would further stimulate the cell to use another line of defense against radicals, namely the overproduction of carotenoids (Figure 1D).

A morphological and physiological evolution including progressive degradation of the chlorophylls and speedy accumulation of carotenogenesis were observed by microscopic observation. The cells during this stress phase cultivation were characterized by apparent change both structurally and biochemically. After 9 days of cultivation, the small green cells were gradually transformed into large orange cells as shown in Figure 1C. This shift in color is due to the degradation of photosynthesis pigments, which are those that function within the photosynthetic machinery, and the accumulation of the secondary carotenoids. The distinctive colors of carotenoids, yellow, orange and red, are related to the presence of a number of conjugated double bonds in a polyene chain that functions as a chromophor ?.

**FIGURE 2:**
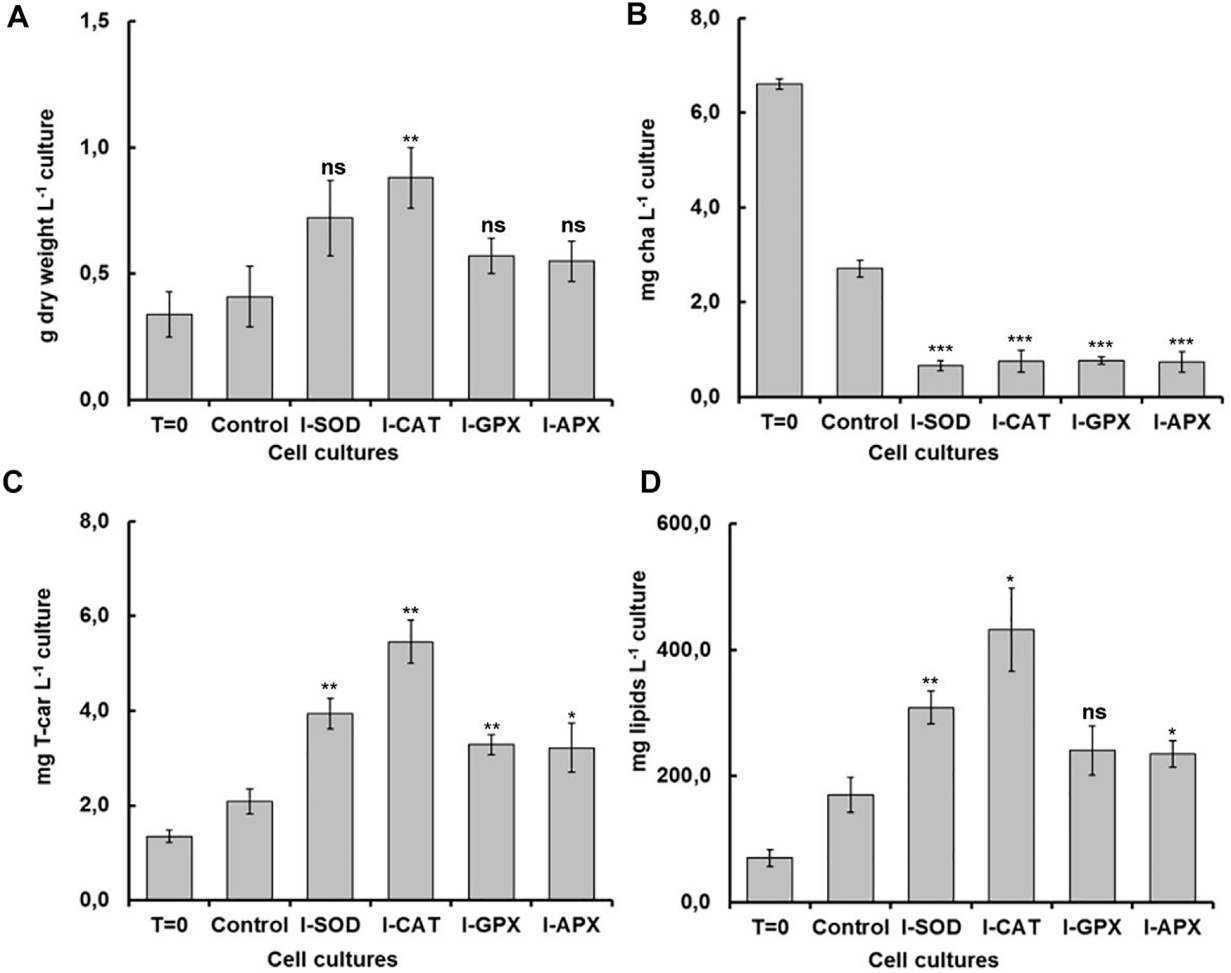
Comparison of dry weight, chla, T-car and lipid concentrations at inoculation (T = 0) and termination (day 9) during the carotenogenesis process for all cultures. **(A)** is dry weight concentration in g L^−1^ of culture. **(B)** is chlorophyll concentration in mg L^−1^ of culture. **(C)** is total carotenoids concentration in mg L^−1^ of culture **(D)** is lipid concentration in mg L^−1^ of culture. *, **, ***, and ns represent statistical significance at 0.05, 0.01, and 0.001, and not significant, respectively.

As shown in Figure 2A, the dry cell weight density at the start of the cultivation was 0.34 ± 0.09 g L^−1^ and increased up to 0.88 ± 0.12 g L^−1^. The highest content was obtained in the I-CAT culture followed by I-SOD, I-GPX, I-APX and finally the control culture with concentrations of 0.72 ± 0.15, 0.57 ± 0.07, 0.55 ± 0.08, and 0.41 ± 0.12 g L^−1^ respectively. The I-CAT culture was significantly different from the control (based on *t*-test) in terms of biomass levels. This biomass increase was due to the accumulation of the different metabolites inside of individual cells rather to cell division. This result surprised us in that the cell cultures with enzyme inhibitors accumulated more biomass than the controlled cell culture.

### 3.2 Photosynthetic pigments degradation

**FIGURE 3:**
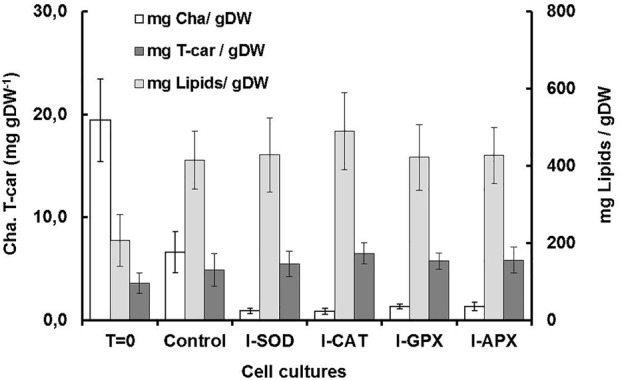
Comparison of dry weight, chla, T-car and lipid concentrations at inoculation (T = 0) and termination (day 9) during the carotenogenesis process for all cultures.

Carotenogenesis cultivation resulted in a quick decrease of photosynthetic pigments. As shown in Figure 2B, chlorophyll a had decreased from 6.61 ± 0.11 mg L^−1^ culture at the start of carotenogenesis to 0.76 ± 0.23, 0.66 ± 0.11, 0,77 ± 0.08, and 0.74 ± 0.21 mg L^−1^ for the cultures of I-CAT, I-SOD, I-GPX, I-APX respectively. The lowest decrease was obtained for the control culture with a concentration of 2.71 ± 0.18 on day 9. The chlorophyll levels of all cultures with enzyme inhibitor added were significantly different from that of the control culture (based on *t*-test). The result was even more remarkable when chlorophyll a content was normalized on biomass (Figure 3). A similar degradation trend was obtained for the chlorophyll b with an initial concentration of 3.91 ± 0.07 mg L^−^1, which reached almost zero on the last day of the culture of this phase for all cultures (data not shown).

The accumulation of carotenoids for all cultures was observed, accompanied by degradation of the chlorophyll content (cha and chb). The orange color is an indicator of the accumulation of secondary carotenoids, in particular canthaxanthin, which is the main secondary carotenoid accumulated by this algal strain during the carotenogenesis process Grama et al. (2014a). Unlike primary carotenoids which are mainly localized in the thylakoid membrane, secondary carotenoids are mainly localized in cytosol vesicles Park et al. (2008). Similar results have been obtained by several studies during the process of carotenogenesis. The reduction of chlorophyll content might be an indicator of strong oxidative stresses Toppi et al. (2004). Under such situations, the carotenoid acts as a protective cellular barrier as well as an antioxidant to quench or remove free radicals and minimize the damage on cell membranes and DNA Czerpak et al. (2006). Carotenoids can provide protection against ROS, either by preventing their formation or by acting as an antioxidant that inactivates them. The quenching effect of carotenoids is due to their polyene structure of conjugated double bonds ?. It was also implied that the photoprotective properties of carotenoids strongly depended on their chemical characteristics Domonkos et al. (2013).

Under normal growth conditions, chlorophylls and carotenoids are produced in chloroplasts in a quantitatively and qualitatively coordinated way. Under stressed conditions, the balance of pigment synthesis shifts toward carotenogenesis and the ultrastructure of the plastids is also modified, accompanied by degradation of the chlorophylls Rabbani et al. (1998). The ratio of chlorophylls to carotenoids is a major factor in preserving the integrity of the photosynthesis system. Therefore, a metabolic balance between carotenoid biosynthesis and catabolism is required to sustain normal growth physiology, especially in photosynthetic cells Beisel et al. (2010). In addition, for photosynthesis, both carotenoids and chlorophylls are essentially connected to peptides in order to produce pigment–protein complexes in the thylakoid membrane Takaichi (2011). Furthermore, carotenoids are fundamental for the assembly and preservation of photosystem II (PSII) and may contribute to electron transfer reactions in this system ?. Other studies indicate that the loss of chlorophyll content may be due to peroxidation of chloroplast membranes Li et al. (2006) and such reduction in chlorophyll levels occurs under increased oxidative stress in microalgae, resulting in less releasing of ROS Cirulis et al. (2013).

### 3.3 Lipids accumulation

As shown in Figure 2D, the lipid concentrations increased during carotenogenesis in all cultural mediums. At the start of carotenogenesis, the initial concentration of lipid was 70.35 ± 13 mg L^−1^, and it reached up to 431.58 ± 66 mg L^−1^ by day 9. The highest value was obtained in the I-CAT culture followed by I-SOD, I-GPX, I-APX and finally the control culture with concentrations of 308.64 ± 26, 240.69 ± 39, 234.81 ± 21, and 170.32 ± 25 mg L^−1^ respectively. The lipid levels of I-SOD, I-CAT and I-APX cultures were significantly different from that of the control (based on *t*-test). The same tendency of lipids increasement was obtained when lipids content was compared on dry cell mass basis. All cultures with enzymatic inhibitors showed higher lipid content compared to the control culture without enzymatic inhibitors added (Figure 3).

Lipid accumulation is another major physiological and biochemical reorientation of cells during stressed phase cultivation. For the carotenogenesis process, the formation of liposomes capable of accumulating lipids and sequestering secondary carotenoids was thought to be a factor shifting the metabolic flux toward secondary carotenoid biosynthesis Solovchenko (2013). Under nutritional stress, the accumulation of lipids in microalgae was enhanced with the formation of triacylglycerol (TAG) as the principal component Du and Benning (2016). Although the formation of liposomes which serve as deposits of secondary carotenoids may occur in the absence of secondary carotenoid biosynthesis, the induction of carotenogenesis is not feasible without such structures ?. Under our culture conditions, the accumulation of lipids was variable in each culture. The highest content was obtained in the I-CAT culture followed by I-SOD, I-GPX, I-APX and finally the control culture. Similar results have been obtained by several studies during the process of carotenogenesis Solovchenko (2013); Grama et al. (2014b). Numerous studies have shown the impact of nutrients on the accumulation of lipids and the decrease of proteins due to nitrogen limitation Wang et al. (2019). This similar phenomenon was also observed in our experiments.

### 3.4 Secondary carotenoids accumulation

As shown in Figure 2C, at different levels and according to the different enzyme inhibitor added, the carotenoid concentrations increased during carotenogenesis in all culture media, compared to the control culture. At the start of carotenogenesis, the initial concentration of T-Car was 1.35 ± 0.13 mg L^−1^, and it reached up to 5.46 ± 0.45 mg L^−1^ by day 9, which was the highest obtained in the I-CAT culture and followed by I-SOD, I-GPX, I-APX and finally the control culture with concentrations of 3.94 ± 0.32, 3.29 ± 0.21, 3.22 ± 0.51, and 2.08 ± 0.26 mg L^−1^ respectively. The levels of total carotenoids of all cultures with enzyme inhibitors added were significantly different from that of the control (based on *t*-test). The same tendency of carotenoids accumulation was obtained when carotenoids was compared on content per unit of dry cell mass basis. All cultures with enzymatic inhibitor showed higher carotenoids content compared to the control culture (Figure 3).

The content and accumulation of secondary carotenoids can vary, depending on the antioxidant enzyme inhibited during the carotenogenesis phase and also depending on the type and concentration of the inhibitors added to the cultures. In addition to that, the response of each algal strain to a given inhibitor can vary, depending on its physiology and the cellular organization of the cells. Therefore, obtaining or optimizing cultures with a high carotenoid productivity was beyond the scope of our study. Our main objective was to study the effect of inhibition of antioxidant enzymes on the accumulation of secondary carotenoids.

In our culture conditions, the highest carotenoid concentration was obtained in the I-CAT culture with a concentration of 5.48 ± 0.45 mg L^−1^. This concentration presents an increase of 2.6 times of the concentration obtained in the control culture. It is noted that catalase is unique among H_2_O_2_-degrading enzymes in that it degrades H_2_O_2_ without consuming cellular reducing equivalents. Hence, catalase supplies the cell with a highly energetically efficient mechanism to eliminate hydrogen peroxide. Consequently, once cells are subjected to energy stress and generate H_2_O_2_ rapidly by catabolic processes, the H_2_O_2_ is degraded by catalase in an energy-efficient way. This should result in a net gain of reducing equivalents and therefore cellular energy Mallick and Mohn (2000).

The carotenoid content obtained was correlated with the lipid content obtained. This has already been reported by other studies which have shown that there is a synchronization of the overproduction of lipids and carotenoids during the carotenogenesis process in microalgae. Rabbani et al. (1998) firstly reported the interdependence between the formation of lipid bodies and the formation of carotenoids in 1998. When the synthesis of triacylglycerol was blocked, the overproduction of *β*-carotene was also inhibited. During overproduction of *β*-carotene, no up-regulation of phytoene synthase or phytoene desaturase was observed on the transcriptional or translational level, whereas at the same time acetyl-CoA carboxylase, the key regulatory enzyme of acyl lipid biosynthesis, was increased, at least at enzymatic activity levels. The authors concluded that, under normal conditions, the carotenogenic pathway was not fully active and might be significantly enhanced by the availability of lipid plastids, thus providing a site-specific sink within the cells for the final carotenoid product of the biosynthesis process Rabbani et al. (1998).

### 3.5 Effects of inhibitor addition

**FIGURE 4:**
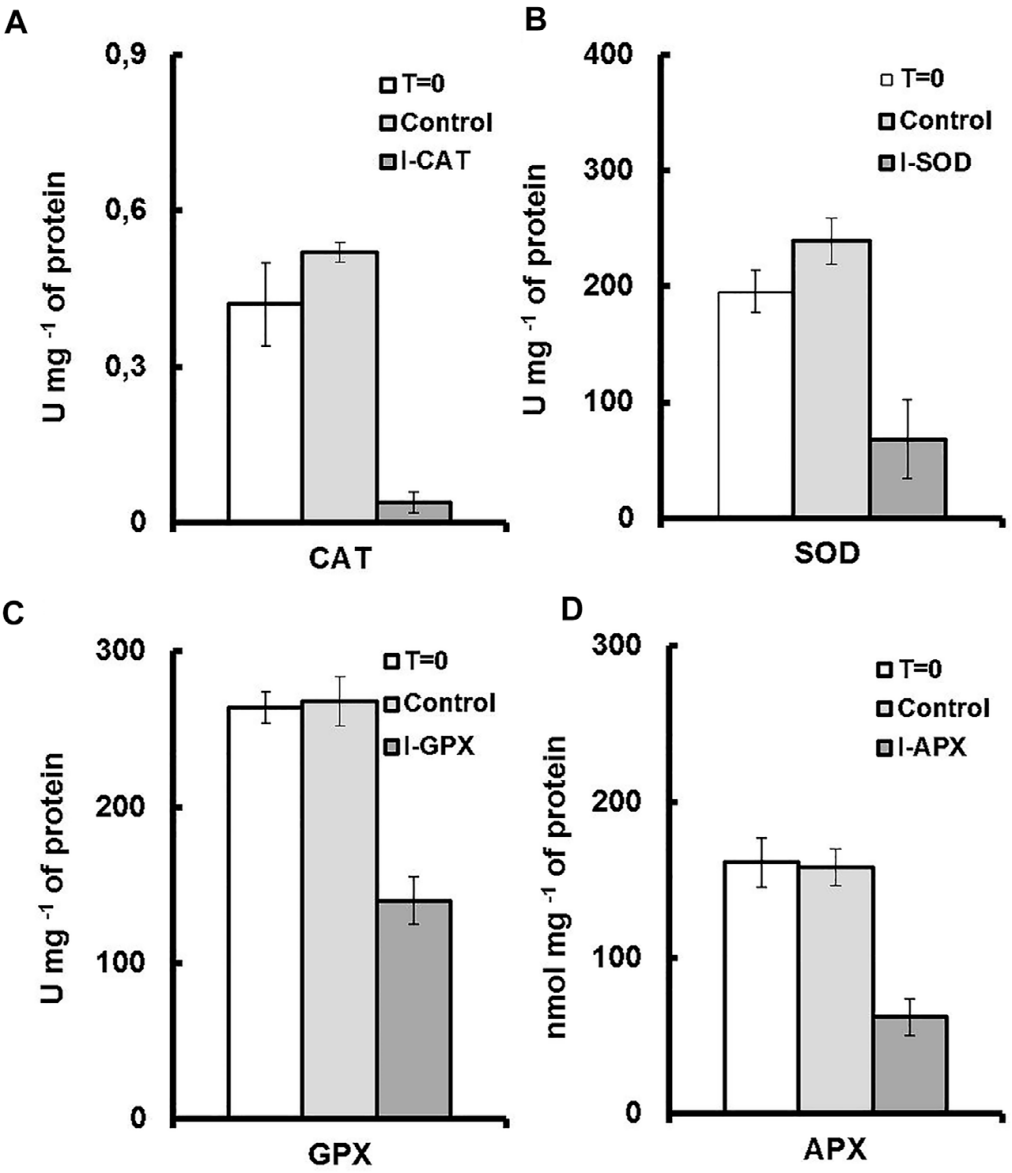
Enzyme activities for each culture medium. T = 0 represents the enzyme activity on inoculation. Control represents the enzymatic activity of the control culture on termination. I-enzyme, represents the enzymatic activity of the culture on termination. **(A)** is CAT activity. **(B)** is SOD activity. **(C)** is GPX activity **(D)** is APX activity.

**FIGURE 5:**
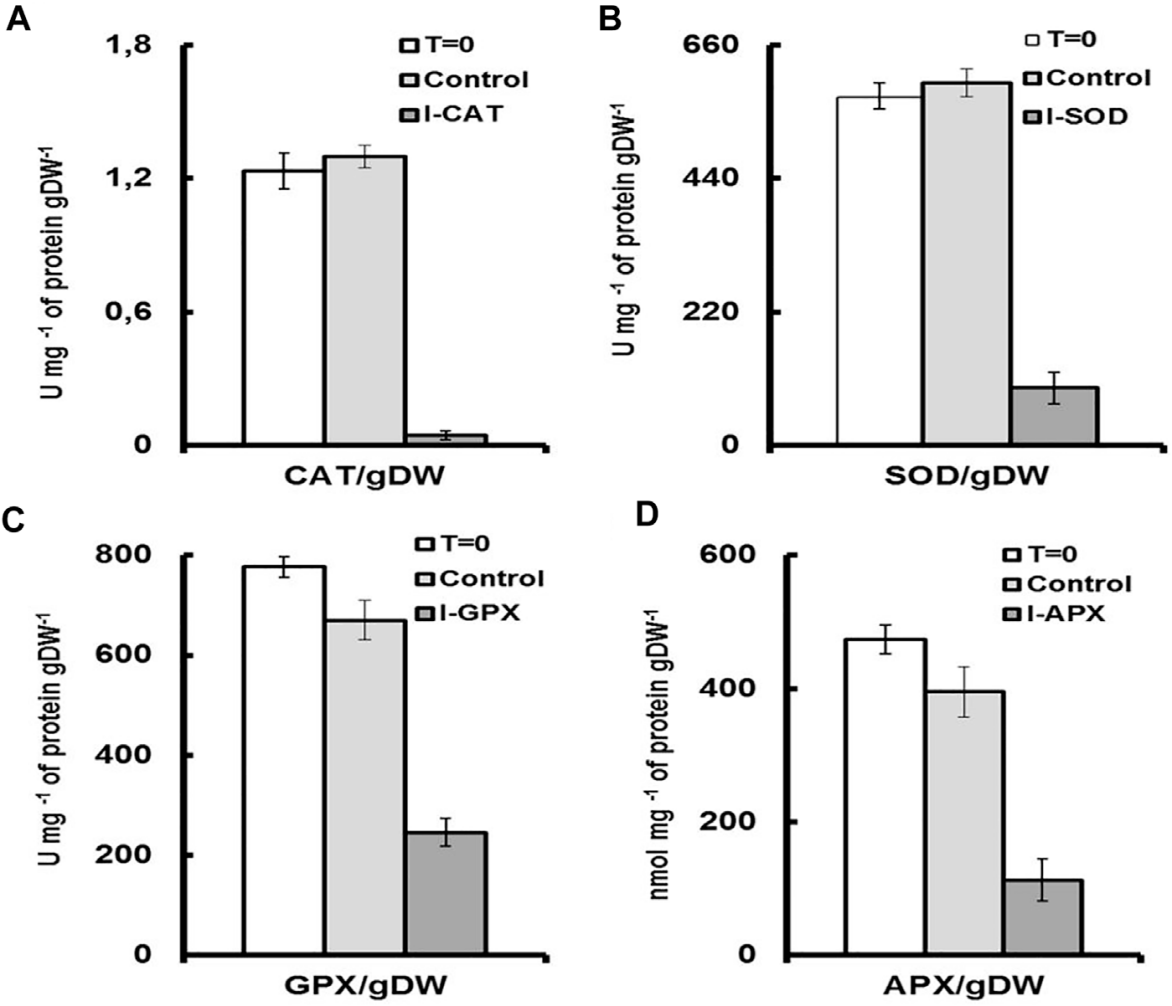
Enzyme activities for each culture medium per unit of dry cell mass. T = 0 represents the enzyme activity on inoculation per unit of dry cell mass. Control, represents the enzymatic activity of the control culture on termination per unit of dry cell mass. I-enzyme, represents the enzymatic activity per unit of dry cell mass of the culture on termination. **(A)** is CAT activity. **(B)** is SOD activity. **(C)** is GPX activity **(D)** is APX activity.

Although numerous exogenous factors might induce oxidative stress in microalgae, most studies have focused on high light and nutrient deprivation to stimulate carotenogenesis Cirulis et al. (2013). In our culture conditions, the activities of the antioxidant enzymes were modulated by addition of different inhibitors. The degrees of inhibition were different for the four targeted enzymes. As shown in Figure 4A, a decrease in the activity of 91% was observed for CAT enzyme followed by 65%, 61%, and 47% for the enzymes SOD, APX and GPX, respectively (Figures 4B–D), the values of which were normalized to total soluble protein in cell culture. The result was even remarkable when enzyme activity was compared on the basis of unit dry cell mass (Figure 5). A decrease in the activity of 96% was obtained for CAT enzyme followed by 84%, 76%, and 68% for the enzymes SOD, APX, and GPX, respectively. In contrast to this, for the control culture which does not contain any enzyme inhibitors, an increase of 19% in activity was obtained for the CAT enzyme and also an increase of 18% for the SOD enzyme (Figures 4A,B). While for the GPX and the APX enzymes, the levels of enzyme activity were almost unchanged (Figures 4C,D). Nevertheless, when enzyme activity was compared on unit dry cell mass (Figure 5), a decrease in the activity of 20% and 16% for both APX and GPX, respectively was obtained, comparing with the starting cell culture.

Plant cells have evolved defensive mechanisms to combat the danger posed by the presence of ROS, which include several enzymatic and non-enzymatic mechanisms. The antioxidant enzymes include mainly superoxide dismutase, catalase, glutathione peroxidase, ascorbate peroxidase. Non-enzymatic mechanisms include carotenoids, ascorbic acid, tocopherols, flavonoids, and other compounds Mallick and Mohn (2000); Park et al. (2008), each of which has its own operating conditions. For an example, there are generally three main characteristics affecting carotenoids to scavenger free radicals: the structure of carotenoids, and the redox potential of carotenoids and the polarity of the medium Jomova and Valko (2013). SOD is present throughout almost all cellular compartments; APX is found in chloroplasts, cytosol, mitochondria, apoplast, and peroxisomes, and GPX and CAT in peroxisomes Mittler (2002). The existence of the ascorbate-glutathione cycle within most of the cellular compartments investigated to-date, as well as the high affinity of APX for H_2_O_2_, implies that this cycle has a critical function in regulating ROS levels within these cellular compartments Mittler (2002).

The intracellular concentrations of ROS are dependent on both ROS production rates and ROS degradation rates by enzymatic catalysis or other mechanisms. The previous strategies for inducing carotenoid production had been focus on stimulating ROS genesis, but our methodology was to increase ROS levels by decreasing the decomposition of ROS by enzymatic catalysis in the various cell compartments. To date there is no study that deliberately alters activity of these enzymes to increase the production of carotenoids, and no work has been reported so far linking the activity of the antioxidant enzymes to the production of carotenoids for the *Dactylococcus* species.

According to the literature, the effect of NaCl on catalase inhibition has already been reported in several works Tejera García et al. (2007) Lichtenthaler (1987). The salt can inhibit catalysis by binding directly to an enzymatic active site or by disrupting the local structure of an active site Park and Raines (2001). For example, the binding of chloride to the heme of catalase was verified by measuring the increase in the Soret absorption peak of the catalase-chloride compound compared to the free enzyme Lichtenthaler (1987). Other authors have proposed that the influence of salt on the enzyme may be due to the binding of water by salts, which changes the structure of the water around the enzyme and thus affects its configuration and activity Lanyi and Stevenson (1969). Catalase contains porphyrin heme active sites that degrade hydrogen peroxide (H_2_O_2_) into water (H_2_O) and oxygen (O_2_) Cirulis et al. (2013). The responses of catalase under salt stress in plants could be species or even strain specific, and the enzymy activity could be enhanced or inhibited Shalata et al. (2001) or no change Fadzilla et al. (1997). In another study, no change in catalase activities over the entire range of 0.05–3.0 mol L^−1^ NaCl salinities was observed for *Dunaliella tertiolecta* Jahnke and White (2003), which was different from our experimental results.

Superoxide dismutase is the first enzyme to operate as an antioxidant defense system in plants as it converts $O_{2}^{-}$ into H_2_O_2_ at a very fast rate Mittler (2002); Rai et al. (2013). SOD is the primary line of defense as well as the unique enzyme able to catalyze this reaction. Therefore, SOD holds a key position within the antioxidant network Janknegt et al. (2007). However, the responses of SOD to environmental stresses depend on the species of the algae and the types of stresses Hong et al. (2008). In addition to that, SOD activity is concentration and time dependent when responding to ROS induced by exogenous stress Cirulis et al. (2013). In the study of Mallick et al. (2002) the authors reported that concentrations of 50–500 mmol L^−1^ of H_2_O_2_ resulted in decreased cell concentrations, growth yields, and chlorophyll concentration for *Scenedesmus obliquus*.

In our study, we have chosen two heavy metals, namely zinc for GPX inhibition and copper for APX inhibition. Although these compounds are essential micronutrients for the metabolism of algae, they are deleterious at high concentrations Ameri et al. (2020). Heavy metals enter algal cells through active transport or endocytosis facilitated by chelating proteins and can disrupt the oxidative balance of algae, including modulation of the activity of GPX and APX Arunakumara and Zhang (2008); Pinto et al. (2003). The characteristics of algal species to accumulate heavy metal ions at varying degrees were also often reported Jordanova et al. (1999).

To inhibit the activity of the enzyme glutathione peroxidase, we used the zinc as an inhibitor. Both the response and toxicity of various algal species to Zn^2+^ were described by Whitton (1971) who reported that algal growth was enhanced at low metal levels and totally inhibited at higher concentrations. In accordance to our study, the GPX activity of *Pavlova viridis* showed the similar trend of evolution when the cells were exposed to zinc. When Zn^2+^ concentration increased, GPX activity decreased gradually and reached about 92.21% of control at 6.5 mg L^−1^. The same effect was reported by the study of Tripathi et al. (2006) with the species of *Scenedesmus* sp. However, for the case of zinc, the inhibition of the growth might not be related to intracellular metal content but rather to its extracellular concentration Wilde et al. (2006). In fact, the suspected mechanism of zinc’s toxic effect might be linked with the cell membrane, where the absorption of calcium required for Ca-ATPase activity in cell division may be affected by zinc concentrations Stauber and Florence (1990). Another study reported that GPX activity was either increased or inhibited, depending on the species, the pollutant, the concentration and the exposure time Lozano et al. (2014).

To target the enzyme ascorbate peroxidase, we used the copper as the inhibitor at a concentration of 10 *μ*M for our experiments. Using the same concentration, Tripathi et al. (2006) reported that Cu^2+^ caused significant inhibition of activity of APX in long-term than in short-term experiments in *Scenedesmus* sp. While, Lozano et al. reported that APX activity did not vary much with 5 and 10 *μ*g L^−1^ of copper in *Cylindrothec aclosterium*, which indicated high level tolerance of oxidative stress for this marine microalga Lozano et al. (2014). Nagalakshmi and Prasad noted that APX activity was enhanced in *Scenedesmus bijugatus* treated with varying copper concentrations (0–100 mM) Nagalakshmi and Prasad (2001).

Comparing the different affinities of APX (*μ*M range) and CAT (mM range) for H_2_O_2_ suggests that they might be two distinct types of H_2_O_2_-removing enzymes. APX could be involved in the fine modulation of ROS for signaling, whereas CAT might be responsible for removing excess ROS under stress Mittler (2002). Catalases are absent from chloroplasts, and thus H_2_O_2_ degradation in chloroplasts might due to ascorbate peroxidase activity Mallick and Mohn (2000).

With the experimental results of the control cultures, we were able to differentiate the effects of enzyme inhibitor addition. An increase of 19% in activity was observed for the CAT enzyme and also an increase of 18% for the SOD enzyme. In accordance with the study of Shi et al. (2017) the same antioxidants enzymes (SOD and CAT) were the leading enzymes with upregulated enzymatic activities under environmental stress. These two enzymes were considered to be the first defense line against ROS damage Ballesteros et al. (2009). Additionally, since CAT and SOD do not depend on the availability of reducing equivalents to perform their roles, they may be unaffected by the redox content of the cells, and their roles remains unaffected during the cultivation process, contrary to the other antioxidant enzymes Mittler (2002).

By comparing with control cell cultures, we were able to notice the effects of adding inhibitors on regulating different antioxidant enzymes during stressed cultivation. The results showed that the inhibition of one of these four antioxidant enzymes resulted in an increase in carotenoids content at variable levels, depending on the enzymes targeted by the inhibition. The result indicated that the massive accumulation of carotenoids could significantly substitute for antioxidant enzymes during the stress phase Park et al. (2008). However, it is important to note that the stress-induced secondary carotenogenesis depends on the species, as well as the profile of secondary carotenoids produced, thus an optimal stress mode might exist for each species of microalgae. It is also important to note that under stress conditions, microorganisms, especially microalgae, can induce the production of isoenzymes to increase their protection. Studies on the several isoenzymes of antioxidant enzyme revealed that there are distinct differences in the responses to oxidative stresses and physiological functions for individual isoenzymes Park et al. (2008). In the well-described study of Park et al. (2008) the authors reported light-induced alterations of antioxidant enzymes by subjecting vegetative green cells of *Haematococcus pluvialis* to high levels of light intensity. Numerous variants of APX, denoted APX1, APX2, APX3, APX4 and APX5 were predicted by native PAGE zymogram. Although the APX5 isozyme was not detected in experiments, a novel band named APXn was detected, which possibly resulted from the biosynthesis of APXn isozyme. Thus, APXn isozyme may play an important role in high light tolerance. Finally, it is noted that comparing with higher plants, the antioxidant defense system to oxidative and other environmental stresses in algae is not well studied at the molecular levels Pinto et al. (2003). This point should be emphasized in order to obtain a comprehensive understanding of the integration of the different lines of cellular defenses against ROS, notably the antioxidant enzymes and the secondary carotenoids.

## 4 Conclusion

Although there are controversial data in the literature, the cellular redox balance of microalgae could be affected by regulating the activity of antioxidant enzymes, depending the particular experimental conditions ?. Individual species could present a different enzymatic activity under the similar environmental conditions. Different microalgal species might exhibit different anti-oxidant enzymatic activities in response to similar environmental conditions. This means that the expected antioxidant responses of microalgal cell culture in any particular location under particular stress will be a combination of factors including the species and even strains Rai et al. (2013). However, our research clearly sheds light on the relationship between the inhibition of antioxidant enzymes and the production of secondary carotenoids in microalga *Dactylococcus dissociatus* MT1. A conclusion can be drawn that modulating the activity of antioxidant enzymes might positively affect the overproduction of valuable secondary carotenoids. This novel and promising approach might allow the development of bioprocesses for carotenoid production from microalgae that avoid the need to deliver high light into dense cell cultures and would therefore be more scalable and efficient than conventional bioprocesses.

## Data Availability

The original contributions presented in the study are included in the article/Supplementary Material, further inquiries can be directed to the corresponding authors.
